# Iron, Human Growth, and the Global Epidemic of Obesity

**DOI:** 10.3390/nu5104231

**Published:** 2013-10-22

**Authors:** Rahul G. Sangani, Andrew J. Ghio

**Affiliations:** 1 Geisinger Medical Center, Danville, PA 17821, USA; E-Mail: rgs4383@gmail.com; 2 Environmental Public Health Division, National Health and Environmental Effects Research Laboratory, US Environmental Protection Agency, Chapel Hill, NC 27599, USA

**Keywords:** ferritin, iron-deficiency anemia, foods, fortified, oxidative stress

## Abstract

Iron is an essential nutrient utilized in almost every aspect of cell function and its availability has previously limited life. Those same properties which allow iron to function as a catalyst in the reactions of life also present a threat via generation of oxygen-based free radicals. Accordingly; life exists at the interface of iron-deficiency and iron-sufficiency. We propose that: (1) human life is no longer positioned at the limits of iron availability following several decades of fortification and supplementation and there is now an overabundance of the metal among individuals of many societies; (2) this increased iron availability exerts a positive effect on growth by targeting molecules critical in regulating the progression of the cell cycle; there is increased growth in humans provided greater amounts of this metal; and indices of obesity can positively correlate with body stores of iron; and (3) diseases of obesity reflect this over-abundance of iron. Testing potential associations between iron availability and both obesity and obesity-related diseases in populations will be difficult since fortification and supplementation is so extensively practiced.

## 1. Introduction

Iron is an essential nutrient utilized in almost every aspect of normal cell function. There are very few organisms that do not have an absolute requirement for this metal. This dependence of life on iron availability is particularly true in aerobic systems which derive energy through an oxidation of hydrocarbons to carbon dioxide with molecular oxygen (O_2_) functioning as a final electron acceptor. Ground state O_2_ exists as a triplet which presents a kinetic block to its reactions with singlet molecules. To overcome this singlet-triplet reaction barrier, transfer of electrons to O_2_ is catalyzed by a transition metal. Iron is positioned in the middle of the first transition series allowing this metal to accept several different oxidation states ranging from 2− to 6+ with the most common being ferrous (2+) and ferric (3+).

Water-soluble ferrous (Fe^2+^) ion was initially present during pre-biotic times and was used by early forms of life [1]. However, Fe^2+^ was effectively removed from the Earth’s crust after its precipitation in oxides and hydroxyoxides following the introduction of O_2_ into the atmosphere by photosynthesis. Ferric ion (Fe^3+^) remained but this is water-insoluble with available concentrations at physiologic pH values approximating 10^−18^ M. Such levels are inadequate to meet the requirements for life and greater concentrations of metal must be procured. Most frequently, metal is obtained by complexation with chelators; oxygen- and/or nitrogen-containing ligands are preferred. While the standard redox potential for the reaction Fe^3+^ + e^−^→Fe^2+^ is +0.77 volt, the redox potential of the reaction ligand-Fe^3+^ + e^−^→ligand-Fe^2+^ can vary from −1 volt to +1 volt as a result of the varying strength of complexation by different ligands [1]. This allows the complexed metal’s participation in the wide range of reactions required in the processes of life during which electrons are transferred.

Subsequently, iron was selected in molecular evolution to support a wide range of biological functions as a result of (1) its interactions with O_2_; (2) its tendency towards complex formation (coordination); and (3) the variability of the redox potential when the metal is chemically complexed. Furthermore, there is simply a greater concentration of iron available relative to other transition metals. However, those same chemical properties which allow iron to effectively function as a catalyst in the critical reactions of life also present a threat via generation of O_2_-based free radicals. Accordingly, life exists at the interface of iron-deficiency and iron-sufficiency.

## 2. Iron Fortification and Supplementation

An insufficient availability of iron has repeatedly challenged human life for millennia. However, during the past 70 years, human intake of iron has been altered to prevent such deficiency (Table 1).

**Table 1:** Interventions into iron homeostasis in the United States.

Intervention	Date
Fortification of food	1941
Fortification of infant formula	1959
Iron supplements to pregnant women	1970s and 1980s
Iron supplements to general population	Recent

First, governments regulated a fortification of food with iron. Following the Nationwide Food Consumptions Survey in the United States (1936–1938), the National Nutrition Conference for Defense presented the first Recommended Dietary Allowances in an attempt to preclude high rates of malnutrition (1941). Despite significant opposition, the United States government initiated a fortification of foods with iron with the intent being to increase the amount of this metal individuals ingest to prevent low metal stores and iron-deficiency anemia. Flour was selected as the major vehicle for iron fortification as white bread provided up to one third of the energy for poorer families. This action would therefore reduce the level of iron-deficiency among the lower socioeconomic classes in which the diagnosis of iron-deficiency was most prevalent. The Food and Drug Administration later allowed for iron fortification of pasta, white bread, corn meal, grits, and white rice with elemental iron, ferrous sulfate, and ferrous fumarate most frequently being employed. Similar actions have been adopted by many nations with wheat flour most frequently being fortified (15 to 60 ppm iron) but milk and condiments have also been used. The worldwide percentage of flour fortification increased to 27% (52 nations) in 2007 [2]. Second, infant formula was fortified with iron. While an artificial substitute for human breast milk was commercially available since 1867, iron fortification of infant formula did not occur until about 1959. In the United States, infant formula is now fortified with iron to an upper limit of 3 mg/100 kcal (about 20 mg/L or 20 ppm) [3]. Such fortification of infant formula is also conducted internationally. Third, there has been widespread provision of iron supplements to pregnant women beginning in the 1970s and 1980s; in the United States, approximately 80% of pregnant women now use them in an attempt to prevent anemia during the last two trimesters (most commonly 60 mg per day). Fourth, iron supplements are now widely used by both men and non-pregnant women with and without physician supervision. In several surveys, such supplements are employed by 40% of the adult population. Provision of supplements to both pregnant women and the general population also occurs internationally.

One result of this increased intake of iron may be a diminished prevalence of iron-deficiency anemia; rates now approximate less than 3% in toddlers, 2% for adolescent girls, and 5% for women of childbearing age in the United States [4]. However, following these interventions to fortify and supplement, it is implausible to consider that human life continues to be positioned at the edge of iron availability. Rather, an overabundance of the metal is predicted in an increased percentage of the population. Regardless of the definition, the prevalence of high iron stores among residents in the United States and in peoples around the world has increased [5,6].

Serum ferritin is most frequently employed as the index of total body iron. Other factors can confound and invalidate the relationship between the iron and serum ferritin. Foremost among these is inflammation which is present in approximately eight percent of a healthy population [7]. As a result of a post-transcriptional control of ferritin expression and some existing equilibrium between pools of iron, metal accessible to cells and tissues also increases following fortification and supplementation [8].

## 3. Body Iron after Fortification and Supplementation

In conditions of dietary iron abundance, the inhibition of iron absorption prevents excess body iron accumulation [9]. Practices of fortification and supplementation were subsequently assumed not to impact the iron status of the normal population; only those with hereditary hemochromatosis were considered to be at risk for iron-overload following fortification. However, there is a significant amount of evidence that iron stores in the normal population can be altered by the practices of fortification and supplementation.

With widespread use of iron supplements by pregnant women, it has been shown that ferritin levels in newborn infants correlated with their mothers’ concentration. This suggests that fetal iron reserves are dependent on maternal iron reserves and, in turn, iron intake [10,11,12,13,14]. In support of this, maternal iron-deficiency anemia during pregnancy compromises fetal iron reserves [10,11,12,13,14,15,16,17]. Serum ferritin concentrations, reflecting stored body iron, are higher in infants of iron-supplemented (but iron-replete) women [18]. Therefore, the iron status of the mother clearly influences that of the newborn infant [10,11,14].

Among children (*i.e.*, six-month-old infants), introduction of iron- fortified formula (12.3 mg/L iron) resulted in significant increases in serum ferritin and other hematological indices in comparison to non-iron fortified formula [19]. This demonstrates that formula can function as an acceptable medium to provide additional iron to growing infants and toddlers. Being a major contributor of dietary iron in developed countries, ingestion of fortified breakfast cereals is strongly correlated with iron status indices amongst the adolescent population [20]. Sequential surveys in urban Danish schoolchildren in the same area (7–17 years) during 1979 and 1986 found increases in overall iron stores [21]. This increase could be attributed to improved dietary iron intake including supplements [21].

In adult populations, dietary intake of iron correlates with levels of body iron in non-anemic, non-deficient adults [5,22]. Dietary factors (*i.e.*, consumption of large quantities of iron rich meat from marine mammals) have contributed to metal accumulation in Greenlandic hunter population [23]. A similar relationship was noticed between native Africans consuming a low iron diet and African Americans in the National Health and Nutrition Examination Survey (NHANES) III who consumed an iron-fortified diet [24]. Continuing to evaluate for an effect of iron fortification and supplementation on metal concentrations in adults, non-pregnant and non-lactating middle age women fed iron-fortified ultra rice (13 mg iron/day) had a significantly higher body iron stores [25]. There is epidemiological evidence showing elevated blood ferritin concentrations among healthy elderly using iron supplements [5]. Comparison of serum ferritin values of US population between NHANES III and NHANES II showed a significant increase in adult females (18 to 44 years of age) and males (18 to 64 years of age); this increase in serum ferritin over an approximately ten-year period may be attributed to the practices of iron fortification, iron supplementation or a high meat intake by the general population [26]. Finally, iron-overload can follow use of supplementation among non-hemochromatotic adults [27].

**Figure 1:**
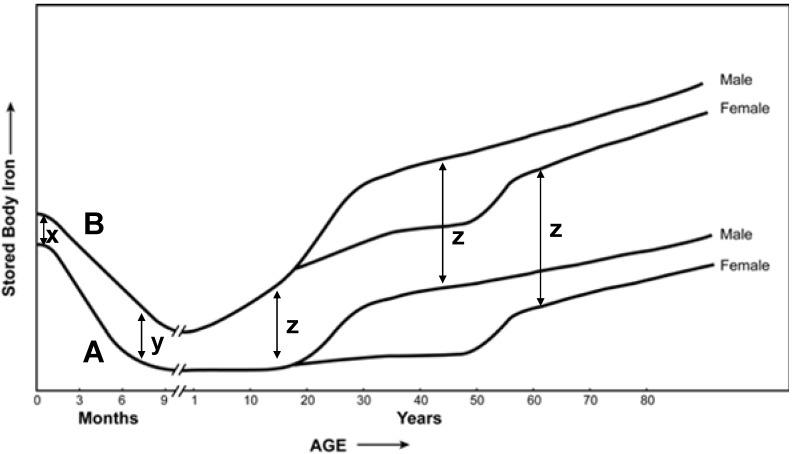
Stored iron in the human. Tracings A and B are proposed to represent stored iron in the human prior to and after, respectively, recent interventions in fortification/supplementation.

Based on this evidence supporting some relationship between dietary intake and levels of body iron, it is proposed that changes in iron fortification and supplementation over the past 70 years have affected an accumulation of the metal in the normal population (Figure 1). It demonstrates two tracings (A & B) showing the relationship between stored body iron and age along with differences in levels of stored iron at various stages of life (*x*, *y*, *z*). Tracing A represents the era prior to the 1940s and tracing B represents current day scenario. As per Tracing A, the newborn had relatively high concentrations of stored iron. During the first few months of life, when milk (which is low in iron) is the primary source of nutrition, this stored metal supported growth of the newborn child. Subsequently, iron stores were mobilized after birth and decreased with nursing of the child. With the introduction of iron-rich solid foods, stored metal concentration increased. Thereafter, an invariable accumulation of iron continued throughout life. Currently (Tracing B), it is expected that body iron stores in the newborn are significantly greater than those previously measured as a result of routine iron supplementation of pregnant women (*x* denotes the difference in iron stores between newborns before and after efforts of fortification and supplementation). While these stores decreased in the first months of life prior to the numerous reported efforts of fortification and supplementation, such loss is now likely to be much less (*y* denotes the difference in iron stores between babies at the end of nursing before and after efforts of fortification and supplementation) as a result of fortified infant formula. Finally, during the remainder of life, body iron stores almost certainly increase at a more rapid rate than previously observed as a result of iron fortification of food and routine use of supplements (*z* denotes the difference in iron stores between adults before and after efforts of fortification and supplementation), comparable in male and female.

## 4. Iron, Growth and Obesity

Over the past several decades, the world has experienced an epidemic of obesity. Five hundred million of the world’s population is now considered to be either overweight or obese and more people are dying from complications of overnutrition than of starvation. The three main determinants of obesity are genetic predisposition, disruption in energy balance, and socio-environmental factors. The genetic pool changes slowly and cannot account for the rapid increase in obesity prevalence. Overall energy intake has stabilized or even slightly reduced. Therefore, dietary factors other than caloric intake have been implicated in this epidemic. It is proposed that increased iron availability resulting from changes in fortification has contributed to the epidemic of obesity.

Cell and molecular pathways of iron potentially impacting growth and obesity have been identified. Iron is an absolute requirement for cell proliferation and cells are unable to progress from the G1 to the S phase of the cycle without it [28,29]. A deficiency of iron leads to apoptosis and cell death [30]. One protein pivotal in cell proliferation is ribonucleotide reductase in which iron is critical for activity [31]. Ribonucleotide reductase is the rate-limiting enzyme involved in the conversion of ribonucleotides into deoxyribonucleotides (dNTPs) for DNA synthesis [32]. Iron chelation provides a mechanism to inhibit the activity of this iron-containing protein. While traditionally it was thought that the anti-proliferative effect of diminished iron availability was solely related to the inhibition of ribonucleotide reductase, there is now growing evidence that this is not the only molecular target. Iron coordinates the progression of the cell through the discreet phases of the cell cycle by affecting the expression of several other molecules including the cyclins, cyclin-dependent kinases (CDKs), cyclin-dependent kinase inhibitors (CKIs) and the tumor suppressor protein p53 [33,34]. Studies have shown that iron availability affects the expression of these proteins critical for cell cycle progression [29,35,36,37,38,39,40]. By altering the expression and/or function of these molecules, iron enhances cell growth. Subsequently, this metal is an absolute prerequisite for *in vitro* cell culture [41]. Transferrin (and lactoferrin) modulate the proliferation of cells but the efficacy depends on their saturation with iron [42]. Iron-saturated forms of transferrin (and lactoferrin) stimulate cell proliferation while the chelator alone suppresses cell growth. In support of the pivotal role of the metal, iron compounds can replace transferrin required for cell proliferation and growth [43,44] and iron depletion by chelators results in cell cycle arrest and programmed cell death or apoptosis [45,46,47,48].

In humans, research confirms increased growth among humans provided greater amounts of iron. Mother’s anemia and/or low serum iron is associated with a small infant size [49,50,51,52]. Newborns of non-anemic mothers supplemented with iron can show greater birthweights (and elevated serum ferritin concentrations) [53]. In anemic children, iron supplementation increases growth [54,55,56,57,58,59]. However, iron status can similarly affect growth in non-anemic and non-deficient children with increased availability of the metal leading to greater growth [60]. Iron status of the infant and older children is a limiting factor for growth even in the absence of deficiency [61]. Non-anemic adolescent girls gain weight with provision of iron [62]. Supplementation of iron produced a significantly greater weight gain in adolescent girls not defined to be anemic or iron-deficient [63]. In contrast, decreased iron availability (e.g., use of iron chelators) can diminish growth [64]. Finally, among healthy adults without either anemia or deficiency, body mass index (BMI) and other indices of body fat correlate with serum ferritin reflecting total body stores of this metal [5,65].

Among foreign-born immigrants in the United States, analysis reveals a significant relationship between the prevalence of overweight and obesity and duration of stay in this country. The increase in BMI was positively associated with serum ferritin supporting an effect of increasing iron availability on growth [66].

## 5. Diseases of Obesity and Iron

Obesity is considered a risk factor for numerous disorders which include the metabolic syndrome and diabetes, cardiovascular disease, cerebrovascular disease, and cancer. Increased availability of iron is recognized as a determinant in their pathogenesis. Therefore, it is proposed that these diseases, comparable to obesity, are associated with increased iron availability.

*Metabolic syndrome and diabetes*. Patients with metabolic syndrome have elevated iron stores [67]. In a cross-sectional study of 6044 adults, serum ferritin levels were associated with the presence of metabolic syndrome [68]. In other investigation, high ferritin levels predicted an increased prevalence of metabolic syndrome at baseline and the incidence at the end of the six-year follow-up period [69]. In addition, the serum concentration of this storage protein correlates with components of the syndrome including hypertension and dyslipidemia [70,71,72,73,74,75].

Epidemiological investigation has reported an association between iron overload in humans and peripheral insulin resistance. Among 1013 middle-aged men, those with higher levels of serum ferritin had greater insulin and glucose levels [76]. Among 1277 adults, serum ferritin level was an independent predictor of an increase in serum insulin level at the end of three years [77]. Relative to women with normal glucose tolerance, those with impaired glucose tolerance had higher serum ferritin levels [78]. Mean ferritin concentrations were higher among women with gestational diabetes compared to those without [79,80]. Two prospective studies also demonstrated an association between serum ferritin levels and gestational diabetes [81,82]. Among pregnant women, iron-supplement users showed significantly higher values of fasting glucose as well as pre-pregnancy BMI, actual BMI, waist circumference, and blood pressure [83].

Comparable to metabolic syndrome and insulin resistance, diabetes is associated with indices of iron homeostasis. Individuals with type 2 diabetes had higher levels of serum ferritin and non-transferrin bound iron relative to healthy controls [84]. Men with high iron stores were 2.4 times more likely to develop type 2 diabetes compared to men with lower stores [85]. Studies have indicated the association between dietary iron and type 2 diabetes risk [86,87,88]. Iron supplementation increased glucose impairment utilization in mid-pregnancy [83]. Diabetic complications, including microvascular and macrovascular disorders (neuropathy, retinopathy, nephropathy, and cardiomyopathy) and vascular dysfunction (hypertension and arteriosclerosis), may be enhanced in patients with increased serum and tissue iron [89,90].

A potential benefit of iron depletion on insulin sensitivity and/or type 2 diabetes has been demonstrated. Frequent blood donors had better insulin sensitivity and lower ferritin levels compared to non-donors [89,90,91]. An increased number of lifetime blood donations were associated with decreased prevalence of type 2 diabetes in men [89,90,91,92]. Iron chelation therapy with intravenous deferoxamine significantly improved metabolic control with a reduction in blood glucose and glycosylated hemoglobin levels among type 2 diabetics [93].

*Cardiovascular disease*. It has been previously postulated that iron depletion protects against ischemic heart disease and that the difference in the incidence of heart disease between men and women could be explained by differences in levels of stored iron. In women, the risk of heart disease does increase following natural or surgical menopause [94]. Among men, there is an increase in risk of coronary heart disease with elevated iron stores. Men with high body iron stores had a two- to three-fold increased risk of myocardial infarction compared with men with low body iron stores [95]. In 1931 randomly selected men with no symptomatic coronary artery disease at entry, the adjusted risk of acute myocardial infarction with serum ferritin greater than 200 ng/mL was 2.2 fold higher than in those with lower serum ferritin [96]. The odds ratio increased by 0.2 for each 100 ng/mL increase in serum ferritin [96]. Mechanistically, evidence for a participation of iron in atherosclerosis was suggested by an ability of the metal to oxidize LDL [97] and damage endothelial cells [98], the observation of ferritin induction with the progression of atherosclerotic lesions [99], the inhibition of endothelial cell damage after oxidized LDL by chelators [100], and the prevention of endothelial cell dysfunction and vascular smooth muscle proliferation by chelators [101]. In animal models, phlebotomy, systemic iron chelation treatment, or dietary iron restriction reduces atherosclerotic lesion size and/or increases plaque stability [99,102,103].

There were positive associations between myocardial infarction and dietary iron intake [99]. A significant association of iron intake and coronary artery disease (CAD) demonstrated that for each milligram of iron consumed, there was an increase of 5% in the risk of CAD [96]. Intakes of dietary iron, especially heme iron and red meat, were significantly associated with a greater risk of fatal CAD, coronary revascularization, and total CAD in diabetic women [104]. Compared with total iron (heme and nonheme) intake, heme iron has been more consistently associated with increased risk of CAD and cardiovascular mortality and the association between heme iron and CAD risk appears to be more marked in postmenopausal women [105,106,107,108,109].

Changes in iron stores during a five-year follow-up period modified the risk of atherosclerosis with the lowering of iron stores being beneficial and further iron accumulation increasing cardiovascular risk [110]. Furthermore, studies on the effect of blood donation on cardiovascular events support the postulate that iron stores can be associated with coronary artery disease [111,112].

*Cerebrovascular disease*. Evidence supports a participation of iron in cerebral ischemia. Experimental iron overload induced using an iron-rich diet causes larger infarct volumes after permanent middle cerebral arterial occlusion in rats [113]. Similar results were obtained in animals with systemic iron overload after administration of intraperitoneal iron dextran [114]. These results indicate that the severity of tissue injury with cerebrovascular occlusion can be proportional to the size of iron stores.

Asymptomatic carotid atherosclerosis, assessed by duplex sonography, shows a strong correlation with iron stores in men and women [115]. Higher serum ferritin concentrations can be associated with an increased risk of ischemic stroke with concentrations > 200 mg/L more than doubling the risk in post-menopausal women [109]. Increased ferritin concentrations, in both blood and cerebrospinal fluid, have been related to poor outcome in stroke patients [116]. Increased serum ferritin concentrations before treatment also predict prognosis in patients with a higher risk of hemorrhagic transformation and brain edema [117].

The participation of iron in cerebrovascular disease could reflect catalysis of oxidative stress in the cerebral vasculature, acceleration of lipid peroxidation in endothelial cells, or activation of platelets via a protein kinase C mechanism [118,119]. Iron released during cerebral ischemia may also contribute to neuronal injury following the stroke [120]. Observations indicate there is a delayed sequestration of released iron in the brain following stroke allowing the metal to damage tissue [120]. Treatment with an iron-deficient diet reduces neuronal necrosis and improves neurological status in animal models of global and focal cerebral ischemia [121].

*Cancer*. Cohort studies have found that measures of body iron stores or dietary iron intake are associated with an increased risk of cancer and cancer mortality. Cancer risk rises after menopause in women in association with rising iron stores [122]. Recent evidence has suggested that increased body iron stores, as indicated by high percentages of transferrin saturation, may be associated with an increased risk for mortality [123,124]. Persons with a normal iron intake did not have higher cancer rates, even when their transferrin saturation level was high [124]. Among persons with increased transferrin saturation, a daily intake of dietary iron of more than 18 mg is associated with an increased risk of cancer [125]. A causal relationship is suggested by studies showing that blood donation (to reduce total body iron stores) is associated with lower cancer risk [126,127] and that blood transfusion adversely affects cancer outcome [128]. A cohort of patients with hepatitis C who were treated with iron reduction and followed for 12 years had a statistically significantly lower risk of developing hepatocellular carcinoma compared with a demographically similar cohort not treated with iron reduction [129]. A randomized trial of calibrated phlebotomy showed a 37% reduction in overall cancer incidence with iron reduction and reduced cancer specific and all-cause mortality among patients [130].

There is an association of iron with colorectal cancer risk; a cohort of the National Health and Nutrition Examination Survey I showed a positive association between dietary and body iron stores with colorectal cancer risk [131]. Body iron stores were also positively associated with the development of precancerous lesions in the colon, colonic adenoma, or polyps [132,133]. It has been postulated that unabsorbed dietary iron increases free radical production in the colon to a level that could cause mucosa damage. Dietary fiber may diminish risk of colorectal cancer by chelating dietary iron through its phytic acid component [134]. Chronic ulcerative colitis patients may be at an increased risk of developing colorectal cancer, because these patients frequently require iron supplementation to remedy iron deficiency anemia due to the presence of rectal bleeding [135].

Data similarly supports the hypothesis that iron overload is a risk factor for liver cancer [136]. Excessive accumulation of iron in hepatocytes causes hepatocellular injury, which leads to the development of fibrosis, cirrhosis, and hepatoma [137]. The increased risk of developing hepatocellular carcinoma in hereditary hemochromatosis has been associated with hepatic iron overload [138]. In a follow-up study of 162 male patients with chronic liver disease related to hepatitis B virus infection, patients who had ferritin levels greater than 300 ng/mL had a 50% chance of hepatocellular carcinoma developing during the follow-up period compared with a 20% risk of hepatocellular carcinoma for patients with lower ferritin levels [139]. A 20-fold increase in the risk of liver cancer in patients with porphyria cutanea tarda has been reported; these individuals frequently demonstrate iron overload [140]. In hepatocellular carcinoma of the non-cirrhotic liver, results of several studies have pointed to increased hepatic iron content [141,142].

Other neoplasms can demonstrate a relationship with body stores of iron. A low iron diet resulted in a decrease in skin tumor incidence (both papillomas and carcinomas) and number of tumors per mouse, as well as the conversion of papillomas to carcinomas [143]. Using a rat model to study the pathogenesis of Barrett’s esophagus and its progression to esophageal adenocarcinoma, it was shown that iron supplemented rats had significantly higher levels of inflammation, cell proliferation, inducible nitric oxide synthase and nitrotyrosine as well as more tumor in their distal esophagi than did rats that received no iron supplement [144].

Experimental study findings support a carcinogenic role of iron in chemically induced carcinogenesis and demonstrate that iron may initiate and promote carcinogenesis through the production of oxidative stress, facilitation of tumor cell growth, and modification of the immune system.

## 6. Conclusions

Iron-deficiency anemia is no longer a public health problem in many places around the developed world. In these nations, the science underlying recommendations for iron fortification and supplementation should be reviewed and its cessation considered. Iron fortification of foods provided to the general population was stopped in both Denmark and Sweden with no increase in iron-deficiency reported [145,146]. Sufficient, or excessive, supplies of the metal continued to be available to these populations as a result of fortification of infant formula, supplementation of pregnant women by physicians, self-supplementation by large segments of community, and recent changes in diet and food habits [147]. Those donating blood were the only individuals who demonstrated changes in stored metal. Since iron is a toxic element and its accumulation can lead to oxidative stress and elevated risk for numerous diseases, supplements should be reserved for those with anemia rather than widespread measures directed at general populations. Supplement users should be made aware of the amount of iron necessary to satisfy dietary requirements and also be informed of the possible deleterious health effects of high body iron stores. The diets of pets may also need to be assessed since, comparable to humans, they currently have their food fortified with iron and are suffering the same consequences of obesity and obesity-related diseases [148].
